# Prehabilitation exercise therapy ahead of elective abdominal aortic aneurysm repair: A commentary of existing evidence to inform clinical practise

**DOI:** 10.12968/bjca.2023.0078

**Published:** 2023-10-19

**Authors:** Zundus Ali-heybe, Areej Mohamed, Oliver Hamer, James Hill

**Affiliations:** 1 NHS Blackpool Teaching Hospitals NHS Foundation Trust; 2 University of Central Lancashire, Preston, UK; 3 NIHR Applied Research Collaboration - Northwest Coast (ARC-NWC), UK

**Keywords:** Abdominal aortic aneurysm, Post-operative outcomes, Prehabilitation, Surgery, Clinical practise

## Abstract

Abdominal aortic aneurysm (AAA) is a condition in which the abdominal aorta becomes enlarged, posing a risk of rupture and life-threatening haemorrhage. Abdominal aortic aneurysm accounts for a substantial number of fatalities worldwide, with mortality rates of up to 80 percent. Abdominal aortic aneurysms are often asymptomatic and are frequently discovered incidentally during tests for unrelated conditions. Surgery is required for aneurysms exceeding 5.5cm in men and 5cm in women, but post-surgical complications such as intra-abdominal adhesions, limb ischaemia and renal failure are common. There is some evidence showing that exercise, including prehabilitation, may be effective in improving patient outcomes post-surgery. However, there is a dearth of literature that has synthesised existing evidence related to the effectiveness of prehabilitation on patient outcomes post-surgery, and which has expanded upon its implications for clinical practise. This commentary aims to critically appraise the most recent Cochrane review in this area, and expand upon these findings to inform clinical practice

## Introduction

Abdominal aortic aneurysm rupture is accountable for approximately 150,000 to 200,000 annual fatalities worldwide and is associated with mortality rates as high as 80% (GBDS 2018; Kessler et al. 2022). Abdominal aortic aneurysm (AAA) is defined as an abdominal aorta which swells and reaches a diameter that is greater than 3 centimetres (in total), or 1.5 times its normal size (NICE 2020a). Most aneurysms do not cause immediate medical concerns, however once enlarged there is a risk of rupture (Haque and Bhargava 2022). Once ruptured, AAA’s are often associated with haemorrhagic shock, with patients requiring emergency surgery (Moreno et al. 2018). However, the majority of patients with AAA’s are asymptomatic with aneurisms often discovered incidentally during tests for unrelated conditions (NICE 2020a). The incidence of AAA is estimated to be approximately 80,000 per year within the United Kingdom (Sidloff et al. 2014). Notably, incidence is significantly higher in men than women, with a ratio of 6:1 (Gao et al. 2023). Diagnosis of AAA is typically made following physical examination and is often confirmed by abdominal computed tomography, magnetic resonance imaging, or ultrasonography (Aggarwal et al. 2011; Lin et al. 2023).

Although the exact cause of an AAA is often unknown, there are many associated risk factors, including hypertension, high cholesterol, smoking, chronic obstructive pulmonary disease, age, family history and male gender (Gao et al. 2023). There are two main strategies for the management for AAA’s which are, regular screening (for non-observable AAA below 5.5 cm) and surgical repair (NICE 2020a). An aneurysm with a diameter greater than 5.5cm in men and 5cm in women requires surgical repair (including open and endovascular aneurysm repair), but post-surgery complications are common (e.g., intra-abdominal adhesions, limb ischaemia and renal failure) (Haque and Bhargava 2022; Pouncey et al. 2021). There are several strategies to reduce post-surgery complications such as smoking cessation, eating a healthy diet and regular exercise (improving cardiovascular fitness) (NICE 2020a). Although guidelines exist that supports these strategies, evidence specifically focused on the effectives of exercise prehabilitation before AAA surgical repair (to reduce postoperative complications), is limited (NICE 2020d; 2020e). As a consequence, a synthesis of existing evidence is needed to determine the effectiveness of prehabilitation on patient outcomes post-surgery, and to outline what the implications of the evidence are in relation to clinical practise.

This commentary aims to critically appraise the most recent Cochrane review in this area (Fenton et al, 2021), and expand upon these findings to inform clinical practice (Fenton et al. 2021).

## Methods of the Review by Fenton et al, (2021)

A comprehensive search strategy encompassing eight databases was conducted which included the Cochrane Vascular Specialised (CRS-Web), the Cochrane Central Register of Controlled Trials, MEDLINE, Embase, CINAHL EBSCO (Cumulative Index to Nursing and Allied Health Literature), PEDro (Physiotherapy Evidence Database), ClinicalTrials.gov and The World Health Organization International Clinical Trials Registry Platform. In addition, bibliographies of the included studies were searched to identify related articles. Database searches were conducted from inception to July 2020 without any language, or publication status restrictions (Fenton et al. 2021).

Randomized controlled trials and controlled clinical trials examining exercise interventions compared with participants who maintained a normal physical activity for people waiting for AAA repair, were included. Variations of exercise therapy were included that were based in hospital, community, or home settings. Additional trials that paired exercise with additional interventions (e.g., psychological counselling, structured education or behaviour change interventions), were also included. Studies that only included subjects going through emergency repair were excluded. The data was only collected for elective trial participants in cases where there were both elective and emergency repairs (Fenton et al. 2021).

Two independent reviewers conducted study selection, data extraction, and assessment of bias. The review’s primary outcomes included both 30-day (or longer, if reported) mortality following AAA repair; and perioperative and postoperative complications (heart, pulmonary, renal, infection, re-intervention, postoperative haemorrhage). Secondary outcomes focused on length of intensive care unit (ICU) stay, length of hospital stay, number of days on a ventilator, change in aneurysm size pre- and post-exercise and quality of life. A meta-analysis was performed, utilizing risk ratios (RR) and their corresponding 95% confidence intervals (CI), employing both fixed and random effects models (Fenton et al. 2021).

## Results of the Review by Fenton et al, (2021)

Following the screening of 762 articles, the review included four trials with a total of 232 participants (Fenton et al. 2021).

There was a statistically significant reduction in risk of occurrence of cardiac complications (RR 0.36, 95% CI 0.14 to 0.92, GRADE: Low), pulmonary complications (RR 0.49, 95% CI 0.26 to 0.92, GRADE: Very Low) and renal complications (RR 0.31, 95% CI 0.11 to 0.88, GRADE: Low) in adults clinically diagnosed with AAA who received exercise compared to those who received no exercise (low to very low certainty evidence: see table 1 for all outcomes) (Fenton et al. 2021).

There was no evidence of difference in 30-day mortality, need for re-intervention or postoperative bleeding in adults clinically diagnosed with AAA who received exercise compared those who undertook no exercise (very low certainty evidence: see table 1 for statistics) (Fenton et al. 2021).

Although meta-analysis could not be conducted, three studies found no evidence of difference in length of ICU and hospital stay of adults with AAA who received exercise compared to those who did not undertake exercise (very low certainty evidence). One study found no evidence of difference in quality of life of adults with AAA who received exercise compared to those who did not undertake exercise (Low certainty evidence). No studies reported on the number of days on a ventilator (Fenton et al. 2021).

## Commentary

Using the AMSTAR-2 critical appraisal tool, a total of 15 criteria out of 16 were judged to be satisfactory (Table 1) (Shea et al. 2017). From this assessment, the systematic review was judged to be provide a comprehensive summary of the existing evidence.

This Cochrane review highlights that there is a lack of high quality evidence as to whether there is a reduction in post-op complications, postoperative bleeding and 30 days mortality and morbidity for those undergoing prehabilitation (Fenton et al. 2021). However, there is low and very low certainty evidence that adults clinically diagnosed with AAA who receive prehabilitation exercise may have a reduced risk of cardiac complications, pulmonary complications, and renal complications (compared to those who received no exercise) (Fenton et al. 2021). Given the low certainty surrounding these estimates, no direct recommendations to clinical practice can be made regarding prehabilitation for adults with AAA.

In regard to the safety profile of prehabilitation, just one RCT reported adverse events as a secondary outcome, but this was not reported in any detail within the review (Fenton et al. 2021). The study found no significant difference in postoperative bleeding and 30-day mortality between exercising and non-exercising group, which may suggest that these interventions do not place patients at additional risk of harm (Barakat et al. 2016). Furthermore, it is commonly perceived that exercise therapy for cardiovascular conditions is typically safe, but further research is needed to confirm this in adults with AAA (Gommans et al. 2015; NICE 2020c).

The 2019 Clinical Practice Guidelines by the European Society for Vascular Surgery (ESVS) concerning the Management of Abdominal Aorto-iliac Artery Aneurysm recommend the consideration of healthy lifestyle interventions, encompassing exercise and diets, for all patients (Dalman 2019). This advice comes with the caveat of acknowledging the limited supporting evidence. In contrast, the National Institute for Health and Care Excellence (NICE) guidelines do not provide specific directives regarding prehabilitation (NICE 2020b). Nevertheless, they emphasize the importance of offering patients support and information to facilitate secondary prevention of cardiovascular disease, which inherently includes guidance on exercise. In light of the scarcity of conclusive evidence, NICE has identified the need for further research and, as a result, has prioritized Prehabilitation as a key area for future investigation (NICE 2020b).

Although limited evidence exists to support prehabilitation, several publications have provided guidance on how physical activity should be performed for adults with AAA (Charlotte et al. 2013; Ehrman et al. 2020). Recommendation from the British Thoracic Society state that people with an AAA of less than 5.5 cm (with controlled blood pressure), may safely partake in a standard multidisciplinary pulmonary rehabilitation of moderate intensity aerobic training (Ehrman et al. 2020). Adults with an AAA greater than 5.5 cm (deemed not fit for surgery) may engage in pulmonary rehabilitation incorporating mild–moderate intensity aerobic exercise, but should not partake in any resistance training (Ehrman et al. 2020). NHS guidelines suggest that mild to moderate exercise could include activities such as walking, vacuuming the home, making the bed, pushing a lawnmower or low intensity cycling (NHS 2021b). Further to these recommendations, literature suggests that when performing these activities, systolic blood pressure should generally be kept below 180 mmHg, and lower than 160 mmHg in patients at greater risk of dissection or rupture (e.g., women and larger sized aneurysm) (Ehrman et al. 2020). Following surgery, recommendations suggest that patients may perform low intensity physical activity such as a short walk (with frequent resting periods) during the first few weeks, but patients should not engage in resistance training until 6 weeks post-surgery (NHS 2021a). Healthcare professionals may use these recommendations to guide patients with AAA in how they could remain active without increasing risks of harm. However, patients engaged in regular exercise should be monitored frequently to identify signs of aneurysm expansion and risk of rupture (small-sized aneurysms once every 2 years, and medium-sized aneurysms once every 3 months) (NICE 2020b).

### Implications for research

Further research is needed in the form of high quality RCT’s (with large sample sizes) to determine the effectiveness of prehabilitation exercise on postoperative outcomes in people with AAA planned for repair. Further research is also needed to establish a core outcome set to improve the quality of evidence-based knowledge, reduce heterogeneity of outcomes across studies, and increase the quantity of data to improve the pooling of results within meta-analyses.

Studies in this area of research have yet to classify the different types of patients, exercises, length of hospital stays, or number of days in the ICU with ventilator support. Future studies should aim to classify these types and also attempt to differentiate patients with high-burden diseases such as hypertension, diabetes and have risk factors, as these other conditions may impact recovery post-surgery.

### CPD reflective questions

- What factors should be considered when making the recommendation for prehabilitation prior to AAA surgical repair?
- What are the key limitations of existing evidence relating to the effectiveness of prehabilitation on postoperative outcomes in people with AAA planned for repair?
- What other benefits may people with AAA receive from engaging in regular exercise?

## Figures and Tables

**Table 1:** Exercise compared to no exercise for adults with clinically diagnosed abdominal aortic aneurysm deemed suitable for elective repair (Fenton et al. 2021).

Outcome	RR (95% CI)	No of studies (participants)	GRADE (Certainty of evidence)	Comments
30-day mortality	RR 1.33 (0.31 to 5.77)	3 RCTs (n= 192)	Very low	There was no evidence of difference in 30-day mortality in adults clinically diagnosed with AAA who undertook prehabilitation exercise compared those who undertook no exercise.
Perioperative and postoperative complications: cardiac complications	RR 0.36 (0.14 to 0.92)	1 RCT (n= 124)	Low	Prehabilitation exercise may decrease the risk of occurrence of cardiac complications compared to no exercise.
Perioperative and postoperative complications: pulmonary complications	RR 0.49 (0.26 to 0.92)	2 RCTs (n= 144)	Very Low	Prehabilitation exercise may decrease the risk of occurrence of pulmonary complications compared to no exercise.
Perioperative and postoperative complications: renal complications	RR 0.31 (0.11 to 0.88)	1 RCT (n= 124)	Low	Prehabilitation exercise may decrease the risk of the occurrence of renal complications compared to no exercise.
Perioperative and postoperative: need for re-intervention	RR 1.29 (0.33 to 4.96)	2 RCTs (n= 144)	Very Low	There was no evidence of difference in a need for re-intervention in adults clinically diagnosed with AAA who undertook prehabilitation exercise compared those who undertook no exercise.
Perioperative and postoperative complications: postoperative bleeding	RR 0.57 (0.18 to 1.80)	1 RCT (n= 124)	Very Low	There was no evidence of difference in postoperative bleeding in adults clinically diagnosed with AAA who undertook prehabilitation exercise compared those who undertook no exercise.
Length of ICU stay (days)	Not reported	2 RCTs (n= 147)	Very Low	Two studies found no statistically significant difference between the prehabilitation exercise and no exercise groups in length of ICU stay.
Length of hospital stay (days)	Not reported	2 RCTs (n= 212)	Very Low	One study reported shorter hospital stay for the exercise group and two studies reported no statistically significant difference between the exercise and no exercise groups.
Number of days on a ventilator	Not reported	Not reported	Not reported	No study reported this outcome.
Quality of Life	Not reported	1 RCT (n= 53)	Low	One study found no statistically significant difference between adults clinically diagnosed with AAA who undertook prehabilitation exercise compared those who undertook no exercise.

**Table 1:** Critical appraisal using the AMSTAR-2 tool for assessing systematic reviews.

AMSTAR 2 items	Responses
1. Did the research questions and inclusion criteria for the review include the components of PICO?	Yes – The study outlined the participants, intervention, comparator and outcomes in the methods section.
2. Did the report of the review contain an explicit statement that the review methods were established prior to the conduct of the review and did the report justify any significant deviations from the protocol?	Yes – The protocol was registered on the Cochrane Database of Systematic Reviews.
3. Did the review authors explain their selection of the study designs for inclusion in the review?	Yes - Studies included all published, unpublished and ongoing trials
4. Did the review authors use a comprehensive literature search strategy?	Yes - Electronic searches of eight databases were included.
5. Did the review authors perform the study selection in duplicate?	Yes – Study selection was independently conducted by two reviewers.
6. Did the review authors perform data extraction in duplicate?	Yes - Data extraction was conducted by two reviewers.
7. Did the review authors provide a list of excluded studies and justify the exclusions?	Yes - The authors provided reasons for exclusion and listed the studies in an appendix.
8. Did the review authors describe the included studies in adequate details?	Yes – A characteristics of included studies table was available.
9. Did the review authors use a satisfactory technique for assessing the risk of bias in the individual studies that were included in the review?	Yes - Two reviewers independently assessed the methodological quality of the identified trials.
10. Did the review authors report on the sources of funding for the studies included in the review?	Yes – The authors did report funding sources for each study where the data was available.
11. If meta-analysis was performed did the review authors use appropriate methods for statistical combination of results?	Yes - Meta-analysis was conducted with appropriate methods using fixed and random effects models
12. If meta-analysis was performed did the review authors assess the potential impact of RoB in individual studies on the results of the meta-analysis or other evidence synthesis?	Yes - The study assessed the potential impact of bias in individual studies on the results of the meta-analysis within the discussion.
13. Did the review authors account for RoB in individual studies when interpreting/discussing the results of the review?	Yes – The authors discussed the results in relation to the quality of evidence.
14. Did the review authors provide a satisfactory explanation for and discussion of, any heterogeneity observed in the results of the review?	Yes – The authors explored heterogeneity within each meta-analysis.
15. If they performed quantitative synthesis did the review authors carry out an adequate investigation of publication bias (small study bias) and discuss its likely impact on the results of the review?	No – The authors stated that there was an insufficient number of trials to assess reporting bias using funnel plots for any of the stated outcomes.
16. Did the review authors report any potential sources of conflict of interest, including any funding they received for conducting the review?	Yes - The authors reported no competing or conflicting interests.

## Key Points

1. Low to very low-quality evidence suggests that adults clinically diagnosed with AAA who receive prehabilitation exercise may have a reduced risk of cardiac complications, pulmonary complications, and renal complications (compared to those who received no exercise).
2. There is no evidence of difference in 30-day mortality, need for re-intervention, or postoperative bleeding in adults clinically diagnosed with AAA who receive prehabilitation exercise compared those who undertake no exercise.
3. Due to the high risk of bias and low to very-low certainty of evidence, recommendations for the clinical implementation of prehabilitation for adults with AAA cannot yet be made.
4. Further research is needed in the form of high quality RCT’s with large sample sizes to determine the effectiveness of prehabilitation exercise on postoperative outcomes in people with AAA (planned for repair).
